# The effect of lycopene in egg shell membrane guidance channel on sciatic nerve regeneration in rats

**DOI:** 10.22038/ijbms.2020.40228.9525

**Published:** 2020-04

**Authors:** Gholam Hossein Farjah, Samad Mohammdzadeh, Masoumeh Zirak Javanmard

**Affiliations:** 1 Neurophysiology Research Center, Department of Anatomy, Faculty of Medicine, Urmia University of Medical Sciences, Urmia, Iran; 2 Department of Anatomy, Faculty of Medicine, Urmia University of Medical Sciences, Urmia, Iran

**Keywords:** Egg shell membrane, Lycopene, Nerve guidance channel, Nerve regeneration, Rat, Sciatic nerve

## Abstract

**Objective(s):**

Peripheral nerves are commonly damaged. Although the nerve autograft still subsists the gold standard in surgery of nerve injury gaps, it has severe detriment. The egg shell membrane (ESM) is a natural material, which has a great potential in practice. The aim of this survey was to appraise the effect of lycopene and the ESM nerve guidance channel on sciatic nerve regeneration.

**Materials and Methods:**

In this study, 32 male rats were randomized into four groups: sham surgery, autograft, ESM+ dimethyl sulfoxide (DMSO), and ESM+lycopene. One centimeter of sciatic nerve was removed, and the gap was grafted by ESM channel or autograft. The sciatic function index (SFI) was evaluated at days 7, 21, 30, 49, 60 and 90 after surgery. Nerve regeneration and gastroknemius muscle fibers were evaluated at days 30 and 90 after surgery by withdrawal reflex latency (WRL), histology and immunohistology assessments.

**Results:**

At 49, 60 and 90 days after surgery, the mean SFI in ESM+lycopene group was significantly greater than ESM+DMSO group (*P<*0.05). On day 90, the mean muscle fiber diameters and the mean number of myelinated axons in ESM+lycopene and autograft groups were significantly greater than ESM+DMSO group (*P<*0.05). In addition, the mean WRL was significantly lower in ESM+lycopene group than in the ESM+DMSO group 90 days after surgery (*P<*0.05).

**Conclusion:**

The results of this study show that the affirmative effects of ESM+lycopene may be beneficial for treating peripheral nerve damages.

## Introduction

Traumatic peripheral nerve damage is popular, and it may result in sensory and motor dysfunction (1). The operational strategy depends on the area, type and level of injury (2). Many operation methods such as epineural repair are appropriate when the nerve is divided by width (3). But when the distance between the terminal ends of the nerve is increased, operation of a transected nerve is impossible without a graft of nerve guidance channel (NGC) (4). Autograft of nerve is still propounded as the golden standard (5); however, this procedure has some obvious detriments including prolonged operation, formation of scare tissue and neuroma in a donor nerve area (6). Therefore, research to identify an appropriate nerve guidance channel is continued (7). Some materials (natural and synthetic) that have been applied as NGCs contain vessels (artery and vein) (8), collagen (9), and egg shell membrane (ESM) (7). Ideally, NGC requires being biodegradable, biocompatible, flexible, semipermeable, easy making and sterilized, and with long-term storage capability (10). A previous study has shown that ESM is effective as NGCs in animal model (7).

Based on its compound, the ESM has an excellent potential in clinical practice (11). Previous study demonstrated that soluble ESM proteins have applications in tissue engineering (12), and as biological dressing for burn wounds (13). The ESM supports the fetus just as the human amniotic membrane does (14). The ESM is a biopolymer plexus that may have potential usages in biomedicine (15). The chicken egg shell and its membranes are a cheap and great loss substance that exhibit interesting specifications for some potential usages (12). In addition, the ESM maintains albumen and prohibits the influence of bacteria (14). 

The ESM has a great amount of non-toxic bioactive components, as well as properties of humidity maintenance, and biodegradability, which have potential application for clinical, cosmetic, and nanotechnology usages (12). 

Lycopene is a type of carotenoid that is found abundantly in tomatoes and other similar fruits such as papaya, watermelon, and pink guava. It has a high antioxidant capacity (16), as a free radical scavenger (17). These attributes confer protection against cancer of the prostate, breast, lung, bladder, cervix and skin, as well as atherosclerosis and associated coronary artery disease (16), and chronic diseases such as cardiovascular disease (18), and diabetes in kidney (19). Lycopene reduces the risk of cardiac dysfunction by enhancement of the antioxidant situation, decrease of inflammation and inhabitation of myocardial fibrosis signaling molecules (20). It has anti-inflammatory and antioxidant effects (17). In addition, it protects cell membranes from lipid peroxidation as a free radical scavenger, and through stimulation of antioxidant enzyme activity (21) *in vivo* and *in vitro* (22). 

Despite the anti-inflammatory and antioxidant properties of lycopene and the positive effect of egg shell membrane, there is no report about the effect of lycopene on peripheral nerve regeneration in ESM channel. Therefore, in the present study we examined the hypothesis that lycopene in ESM guidance channel enhances sciatic nerve regeneration. 

## Materials and Methods

***Animals***

Thirty two 10-week-old adult male Sprague-Dawely rats (220-260 g) were randomized into four groups, including sham surgery, autograft, ESM+dimethyl sulfoxide (DMSO), and ESM+lycopene (10 mg/kg). The animals were kept in 12L: 12D cycle at a room temperature (22±2 ^°^C) with free access to water and food. The study was approved by the ethical committee of Urmia University of Medical Sciences (approval No: IR.UMSU.REC.1396.311, date 13 Dec 2017). 

***Preparation of the ESM nerve guidance channel***

**Figure 1:**
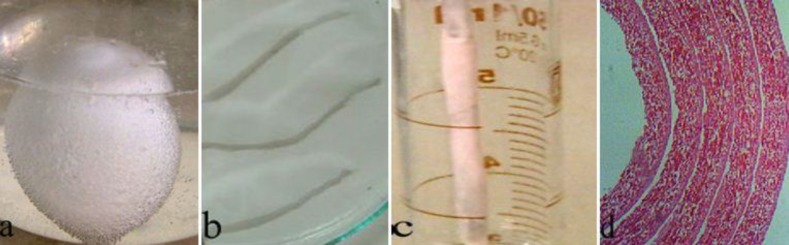
a: The hen eggs were soaked in 4% acetic acid. b: The decalcifying egg shell membranes cut into four pieces. c: The egg shell membrane was spined over the Teflon rod to have a longitudinal orientation. d: Photomicrograph of the ESM nerve guidance channel (Hematoxylin-Eosin stain, ×400). ESM: Egg shell membrane

Preparation of the ESM nerve guidance channel has already been explained in our former study (7). Briefly, four new hen eggs were soaked in 4 % acetic acid for 7 days. Then, the egg shells were spined over the Teflon rod to have a longitudinal orientation. Then, the ESM channel was separated from Teflon rod, and stored in the refrigerator. The ESM channel measured 1.7 mm in inner diameter, 12 mm in length, and wall thickness of 0.5 mm (Figure 1). 

***Surgery procedure***

The rats were anesthetized (ketamine: 100 mg/kg; xylazine: 15 mg/kg,IP). In the sham surgery group, the left sciatic nerve was exposed. In the other groups, a 1 cm nerve segment was removed (proximal to the sciatic nerve bifurcation). In ESM groups, the nerve cutting ends were implanted into the NGC, and sutured to the channel wall (0-10 nylon). The NGCs were filled with DMSO (30 µl) or lycopene (10 mg/kg/bw; prepared up to 30 µl with PBS solution, Sigma-Aldrich Chemie, Munich, Germany) (23). Sterile wax was used to seal the channel to prevent leakage. In autograft, the 1 cm nerve piece was sutured to the nerve cutting ends. Then, the muscle and the skin were sutured. 

***Functional tests***

The rats were appraised one day before operation and in the 7^th^, 21^st^, 30^th^, 49^th^, 60^th^ and 90^th^ days post-operation. Black ink was applied to the plantar surface of the hind limbs. The rats were permitted to walk down the track, impressing its hind limb footprints on the sheath. The footprints of both the hind limbs were applied to calculate the sciatic functional index (SFI) (24). 

***Muscle weight***

After neurological assessment, gastrocnemius muscle was perfectly separated from the bone, and the wet weight of muscle was measured by digital scale (25).

***Histologic evaluation***

At the 30 and 90 days after surgery, the middle segment of the sciatic nerve and gastrocnemius muscle were removed in experimental groups. The samples were fixed in 10 % formalin solution, dehydrated, embedded in paraffin blocks, and cut into 5 µm sections. The nerve cross sections were stained with toluidine blue. Then, the total myelinated fibers were counted. In addition, four microscopic fields in each section were randomly selected. Then, the myelinated fiber diameter and myelin sheath thickness measurements were performed with calibrated eyepiece (26). 

The gastrocnemius muscle cross sections were stained with Hematoxylin-Eosin (H-E). Then, four microscopic fields in each section were randomly selected, and the diameter of muscle fibers was measured with calibrated eyepiece (25). 

***Withdrawal reflex latency***

Thirty and ninety days after surgery, sensory nerve recovery was appraised by evaluating withdrawal reflex latency (WRL). The WRL was determined at the time that the animal withdraws its hind limb paw because of heat. With this intention, the hind limb paw was placed on a hot plate (56 ^°^C), and the time from the beginning of contact to maximum time in the left of hind limb was evaluated. The maximum time for this test was 10 sec to prevent skin injury to the animal paw (27). 

***Immunohistochemistry***

An anti S-100 (Dako, 1:200 dilution) was applied as a sign for Schwann cells. The nerve samples were fixed (4 % paraformaldehyde), embedded in paraffin and cut into 4 µm cross sections. According to the instruction of S-100 staining kit, non-specific immunoreactions were blocked. Then, the samples were incubated in S-100 protein antibody solutions. Horseradish peroxidase-labelled secondary antibody solution was added to the sections, incubated for 15-20 min, washed with PBS, and the results were evaluated under a light microscope. Then, the total number of S100-positive Schwann cells were counted in the cross sections of each sciatic nerve (28).

***Statistical analysis***

Statistical analysis was performed with 95% confidence intervals by using SPSS 16.0 for Windows (Chicago, IL, USA). All values were provided as means±SEM. Statistical analysis was performed by one-way ANOVA followed by Tukey´s *post hoc* test. The differences were considered significant when *P*<0.05. 

## Results

**Figure 2:**
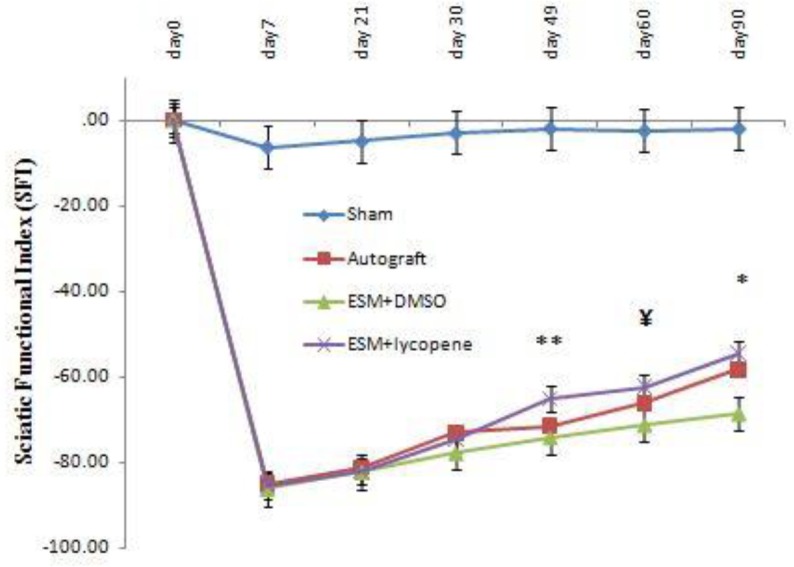
The SFI decreased at 7 days after surgery

The SFI was acutely decreased in experimental groups at day 7 post-operation. On the days 49, 60, and 90 post-operation, the mean SFI were -65.08±2.9, -62.37±1.97, and -58.31±4.98 for the ESM+lycopene group, and -77.15±4.44, -76.95±4.94, and -68.64±7.89 for the ESM+DMSO group, respectively (*P*<0.03). Moreover, the ESM+lycopene and autograft groups were statistically different from the sham surgery group (*P*<0.05). There were no statistically significant differences between ESM+lycopene and autograft SFI values (*P*>0.05) (Figure 2).

**Figure 3:**
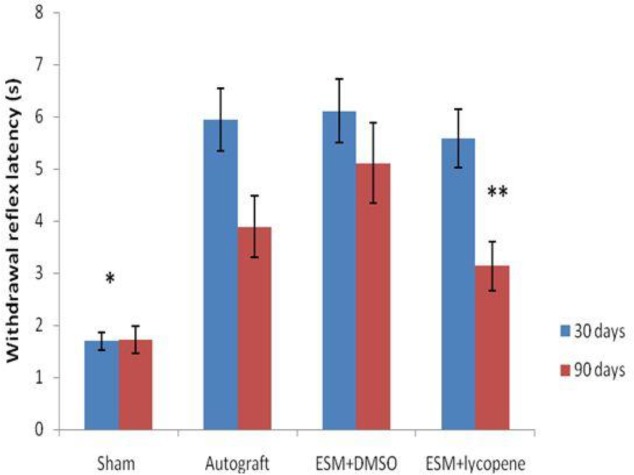
The mean WRL after surgery in experimental groups. * Difference between sham surgery and other groups, 30 and 90 days after surgery (*P<*0.05). ** Difference between ESM+lycopene and ESM+DMSO groups, 90 days after surgery (*P<*0.019). Results are presented as mean±SEM. WRL: Withdrawal reflex latency, DMSO: Dimethyl sulfoxide, ESM: Egg shell membrane

WRL was significantly lower in the ESM+lycopene group than in the ESM+DMSO group 90 days after surgery (*P*=0.019) (Figure 3). 

**Figure 4.**
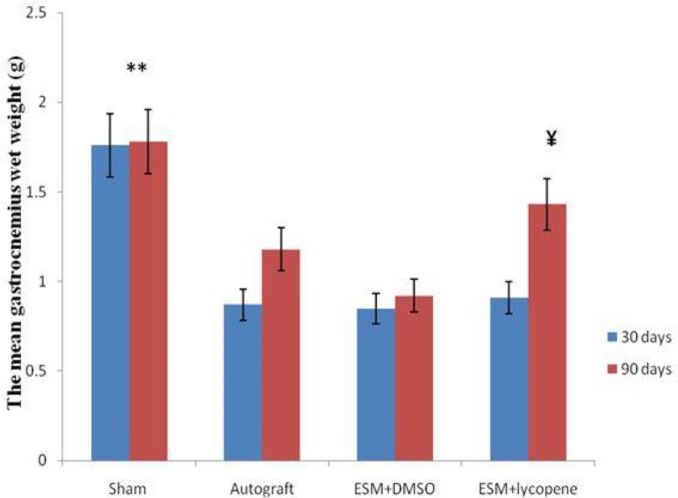
The mean gastrocnemius wet weight (g) after surgery in experimental groups

At 30 days after surgery, the mean weight of muscles in all experimental groups reached its lowest value, but there were not significant statistical differences between them (*P*>0.05). Ninety days after surgery, there were significant statistical differences between the ESM+lycopene (1.43±0.23) and ESM+DMSO groups (0.92±0.27) (*P*<0.001) (Figure 4). 

**Figure 5:**
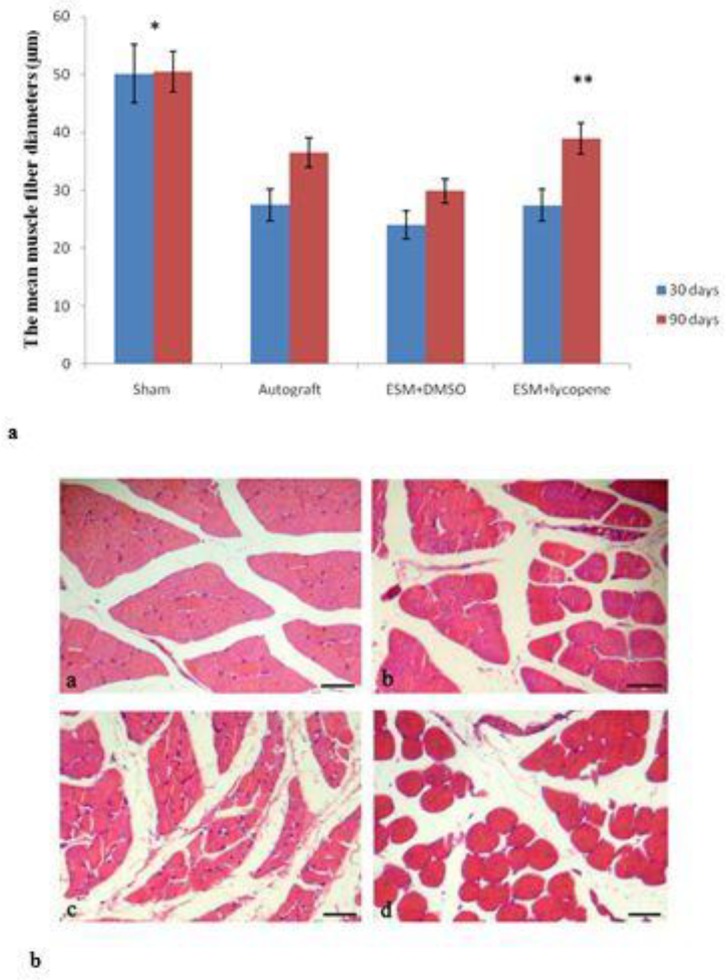
a: The mean muscle fiber diameters (µm) after surgery in experimental groups. *Difference between sham surgery and other groups (*P<*0.01). **Difference between ESM+lycopene and ESM+DMSO groups, 90 days after surgery (*P<*0.001). Results are presented as mean±SEM. b: Cross section of the gastrocnemius muscle (H-E) showing muscle fiber morphology after nerve regeneration. (a) sham surgery, (b) autograft, (c) ESM+DMSO, (d) and ESM+lycopene, 90 days after surgery (Scale bar 50 µm). DMSO: Dimethyl sulfoxide, ESM: Egg shell membrane

Thirty days after surgery, the mean muscle fiber diameters in all groups were lower than sham group, and the differences were statistically significant (*P*<0.01). At 90 days after surgery, the result showed maintenance of muscle anatomy with less fiber atrophy in ESM+lycopene group. The mean muscle fiber diameters in ESM+lycopene group in comparison with ESM+DMSO group showed significant differences (*P*=0.33) (Figure 5). 

**Figure 6.**
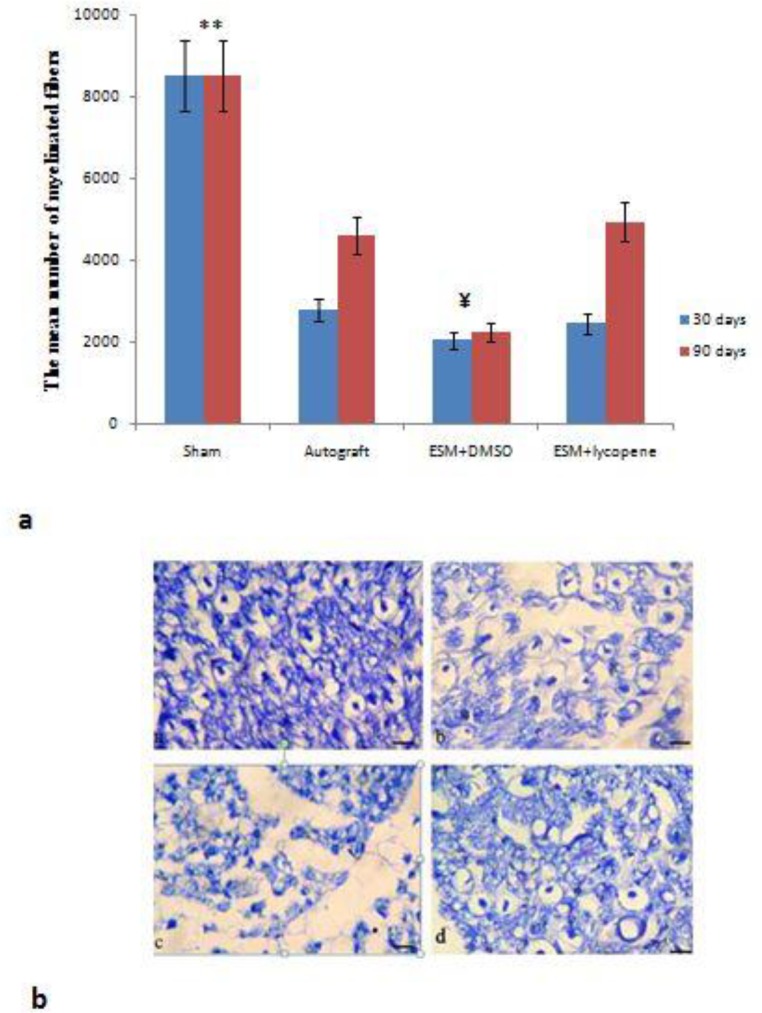
a: The mean number of myelinated fibers after surgery in experimental groups. ** Difference between sham surgery and other groups (*P<*0.001). ¥ Difference among ESM+DMSO, ESM+lycopene, and autograft groups, 90 days after surgery (*P<*0.001). Results are presented as mean±SEM. b: Cross section of the main axis of the regenerated nerve (Toluidine blue stain), showing the myelinated axons after nerve regeneration. (a) sham surgery, (b) autograft, (c) ESM+DMSO, and (d) ESM+lycopene, 90 days after surgery (Scale bar 50 µm). DMSO: Dimethyl sulfoxide, ESM: Egg shell membrane

After cutting the channel, we observed that the nerve tissue was successfully connected to two terminal ends nerve. The nerve cables were centrally placed in the ESM channel. The regenerated axons in experimental groups had lower diameter axons and thinner myelin sheath than the sham surgery group. Thirty days after surgery, in the experimental groups, blood vessels and regenerated axons were apperceived all over the regenerated tissue with a great content of connective tissue around them. Finally, after 90 days, the myelinated axons were more organized in microfascicles with a small connective tissue in the ESM+lycopene and autograft groups, while in the ESM+DMSO group, the regenerated axons were surrounded by abundant connective tissue around them. At 90 days post-operation, the mean number of myelinated fibers in ESM+lycopene group (4940±764.67) was significantly greater than ESM+DMSO group (2276±870.39) (*P*<0.018), but no significant difference was found among ESM+lycopene and autograft (4603±879.16) groups (*P*<0.05) (Figure 6).

The ESM+lycopene group showed siginificantly greater myelinated fiber diameter than ESM+DMSO group at 90 days after surgery (Table 1). 

**Figure 7:**
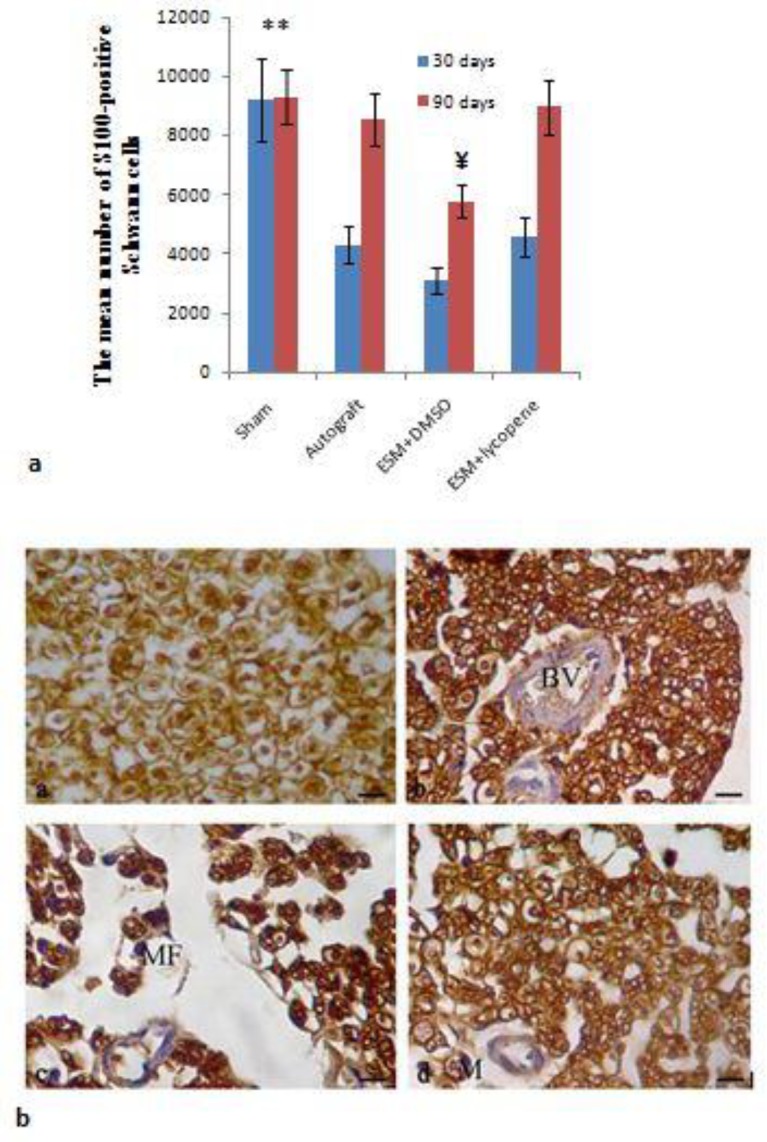
a: The mean number of S100-positive Schwann cells after surgery in experimental groups

At the 30 and 90 days after surgery, distinctly more positive immunoreactivity to S-100 protein was greatly found in the ESM+lycopene and autograft groups, suggesting that Schwann cells existed around the myelinated axons. Figure 7a shows that the mean number of S100-positive Schwann cells in ESM+lycopene group were significantly greater than ESM+SMSO group (*P*<0.05). In addition, in ESM+lycopene and autograft groups, the anatomy of myelinated axons was more alike to those of intact nerve compared to ESM+DMSO group (Figure 7b).

## Discussion

This survey indicated that lycopene in ESM channel increases sciatic nerve regeneration. To the best of our knowledge, this is the first study to evaluate the effect of ESM+lycopene on sciatic nerve regeneration. There are many benefits of using ESM channel, including low cost, and flexibility; besides it, is strong enough to maintain a suture. In addition, the dimensions of the channel are easily controlled (7). 

Our previous study showed that ESM as NGCs enhances peripheral nerve regeneration in animal models (7). The chicken ESM contains collagen type I, III (29), hyaloronic acid (30), and laminin (31). These compounds are important in nerve regeneration (31-34).

In the present study, the SFI was evaluated up to 90 days after surgery, because most of the functional recovery occurred at 14 and 90 days post-operation (35). The parameters in SFI assessment are related to the intrinsic muscle function of the feet (36). Therefore, an increase in SFI in the autograft and ESM + lycopene groups implies an increase in axon regeneration in the hind limb muscles. Our results are in agreement with the finding of previous study that showed in spinal cord injury, administration of lycopene would improve motor impairment in the hind limb of the rat (37).

There is a hypothesis that the functional recovery is a more comprehensive evaluation than histomorphometric procedures in peripheral nerve studies (38). In this study, we apperceived an increase on multiple parameters such as SFI, the number of myelinated fibers, myelin sheath, and muscle fiber diameters in the ESM+lycopene group. The results showed useful effects of lycopene via faster and significant improvement of the nerve function in SFI. The results of a previous study also demonstrated that administration of lycopene may be effective in management of uveitis through inhibiting inflammation and oxidative stress (23). There is an opinion that the SFI is rather comprehensive and valid than histomorphometric procedures in sciatic nerve repair survey (39). 

There are more reasons why it is difficult to compare our results with other studies: 1) chemical combination and structure of the NGC; 2) length of the gap; and 3) no published reports are available for the application of the lycopene in NGCs. 

In the present study, DMSO was used as a lycopene solvent. The results showed that lycopene in the ESM guidance channels added mean number of myelinated axons and myelin sheath thickness in comparison with ESM +DMSO. DMSO permeabilizes cell membranes and it is used as a facilitator in the delivery of molecules into the cell (40). After axonal damage, oxidative stress is known to be one of the great causes of neural injury. It has been shown that antioxidant molecules play a major role in nerve damage and regeneration (41). Hu *et al.* (2017) showed that lycopene administration through increasing total antioxidant levels may have a protective effect on the spinal cord injury (37). Pander *et al.* (2016) showed that lycopene has anti-inflammatory effects (19). Inflammation is necessary for regeneration in the nervous system. However, it has negative effects that prevent improvement (42). The inflammatory cytokines such as interleukin 6 (IL-6), and tumor necrosis factor-alpha (TNF-ɑ) increased in nerve tissue damage. Lycopene decreased IL-6, and TNF-ɑ expressions (20). 

In the present study, a layer of macrophages cold be observed on the surface of the ESM with obvious marks of phagocytosis of the biomaterial. The macrophages produce trophic factors that can enhance nerve regeneration (43). In addition, the macrophages and their released cytokine (interleukin-1β) could also stimulate the secretion of several growth factors in nerve (44).

Goel and Tyagi (2016) showed that lycopene administration increased the number of myelinated and unmyelinated fibers after sciatic nerve ligation (45). In this study, the mean myelin sheath thickness for the ESM+lycopene group was greater compared to the ESM+DMSO, and autograft groups, but the difference was not significant. Many neurotrophic factors and extracellular matrix molecules are secreted by Schwann cells that are effective in the survival of neurons (46). 

In this study, the mean gastrocnemius muscle wet weight and the mean muscle fiber diameters was evaluated. Such that lack of neural innervations of gastrocnemius muscle resulted in a decrease in the muscle bulk (47). In addition, denervation of a muscle increases oxidative stress and consequently reduces skeletal muscle mass. Lycopene, as an antioxidant, helps prevent skeletal muscle loss (48). 

Lycopene adjusts many cellular functions, including cell proliferation, glucose metabolism, cell hypertrophy, and cell apoptosis (49). Our result demonstrated that the mean number of myelinated fibers in ESM+lycopene group were significantly greater than ESM+DMSO group. In addition, the use of lycopene over DMSO has a great positive effect on SFI at 46 days after surgery, and this effect is protected until the end of the experimental period.

In this study, the position of positive reactions to S-100 in ESM+lycopene group was obviously more positive than ESM+DMSO group. This phenomenon demonstrated that the expression of S-100 corresponded with proliferation of Schwann cells in response to peripheral nerve damage (50). 

** Difference among ESM+lycopene with the ESM+DMSO, 49 days after surgery (*P<*0.007). ¥ Difference among ESM+lycopene with the ESM+DMSO, 60 days after surgery (*P<*0.002). * Difference among ESM+lycopene with the ESM+DMSO, 90 days after surgery (*P<*0.03). Results are presented as mean±SEM. SFI: Sciatic functional index, DMSO: Dimethyl sulfoxide, ESM: Egg shell membrane

** Difference between sham surgery and other groups (*P<*0.001). ¥ Difference between ESM+lycopene and ESM+DMSO groups, 90 days after surgery (*P<*0.035). Results are presented as mean±SEM. DMSO: Dimethyl sulfoxide, ESM: Egg shell membrane

**Table 1:** Myelinated fiber diameter (µm), and myelin sheath thickness (µm) in the experimental groups, 30 and 90 days after surgery

**Groups**	**Myelinated fiber diameter (µm)** **30 days 90 days**	**Myelin sheath thickness (µm)** **30 days 90 days**
**Sham surgery**	10.96±1.43^*^ 10.98±0.43^*^	1.42±0.31^*^ 1.43±0.35^*^
**Autograft**	3.73±0.24 5.69±0.58^**^	0.27±0.11 0.58±0.07
**ESM+DMSO**	3.61±0.22 4.99±0.12	0.26±0.04 0.43±0.09
**ESM+lycopene**	3. 91±0.49 6.48±0.29^**^	0.26±0.03 0.56±0.06

* Difference between sham surgery and other groups (*P<*0.01)

** Difference between ESM+lycopene and autograft groups with ESM+DMSO group (*P<*0.05)

DMSO: Dimethyl sulfoxide, ESM: Egg shell membrane

** Difference between sham surgery and other groups, 30 days after surgery (*P<*0.001). ¥ Difference between ESM+DMSO group with ESM+lycopene and autograft groups, 90 days after surgery (*P<*0.01). Results are presented as mean±SEM. b: Immunohistochemical analysis of transverse sections of the regenerated nerve 90 days after surgery. (a) Sham surgery, (b) Autograft, (c) ESM+DMSO, and (d) ESM+lycopene. There was S100-positive Schwann cells. Regenerated nerve fibers including myelinated axons, Schwann cells and blood vessels were present all over the tissue (scale bar 20 µm). MF: micro fascicle, BV: blood vessel, M: macrophage, DMSO: Dimethyl sulfoxide, ESM: Egg shell membrane

## Conclusion

The ESM and lycopene may be beneficial for treating peripheral nerve damages. However, more studies are required to determine the efficiency and mechanisms of lycopene in peripheral nerve regeneration. It is suggested in future studies to investigate the effect of lycopene on nerve regeneration with collagen or laminin as matrix in ESM guidance channel.
